# The PreCardio-study protocol – a randomized clinical trial of a multidisciplinary electronic cardiovascular prevention programme

**DOI:** 10.1186/1471-2261-7-27

**Published:** 2007-09-04

**Authors:** Neree Claes, Nele Jacobs

**Affiliations:** 1 Faculty of Medicine, University Hasselt, Agoralaan Building D, BE-3590 Diepenbeek, Belgium

## Abstract

**Background:**

Cardiovascular diseases (CVD) are the leading cause of death and the third cause of disability in Europe. Prevention programmes should include interventions aimed at a reduction of medical risk factors (hypertension, hypercholesterol, hyperglycemia, overweight and obesity) as well as behavioural risk factors (sedentary lifestyle, high fat intake and low fruit and vegetable intake, smoking). The aim of this study is to investigate the effects of a multifaceted, multidisciplinary electronic prevention programme on cardiovascular risk factors.

**Methods/Design:**

In a randomized controlled trial, one group will receive a maximal intervention (= intervention group). The intervention group will be compared to the control group receiving a minimal intervention. An inclusion of 350 patients in total, with a follow-up of 3 years is foreseen. The inclusion criteria are age between 25–65 and insured by the Onderlinge Ziekenkas, insuring for guaranteed income in case of illness for self-employed. The maximal intervention group receives several prevention consultations by their general practitioner (GP) using a new type of cardiovascular risk calculator with personalised feedback on behavioural risk factors. These patients receive a follow-up with intensive support of health behaviour change via different methods, i.e. a tailored website and personal advice of a multidisciplinary team (psychologist, physiotherapist and dietician). The aim of this strategy is to reduce cardiovascular risk factors according to the guidelines. The primary outcome measures will be cardiovascular risk factors. The secondary outcome measures are cardiovascular events, quality of life, costs and incremental cost effectiveness ratios. The control group receives prevention consultations using a new type of cardiovascular risk calculator and general feedback.

**Discussion:**

This trial incorporates interventions by GPs and other health professionals aiming at a reduction of medical and behavioural cardiovascular risk factors. An assessment of clinical, psychological and economical outcome measures will be performed.

**Trial registration:**

ISRCTN23940498

## Background

In Europe, cardiovascular disease (CVD) is the leading cause of death (1.5 million deaths per year) and is an important source of disability [1-3]. The incidence of coronary vascular events is 1.91 per 1000 population years [4]. It is proven that prevention of CVD risk factors lead to a reduction of CVD[5,6]. Prevention programmes should include interventions aimed at a reduction of medical risk factors (hypertension, hypercholesterolaemia, hyperglycaemia and overweight) and behavioural risk factors (sedentary lifestyle, high fat intake and low fruit and vegetable intake, smoking) for CVD[7,8] The individual contributions of these risk factors to CVD are: high blood pressure (62%), high cholesterol (18%), overweight (13%), low fruit and vegetable intake (11%), physical inactivity (7%), tobacco (12%) and alcohol (4%). The joint contribution of these risk factors is 70–76%[9]. Recent guidelines advise to determine the overall risk on CVD using the SCORE Model, scoring the individual risk in terms of the absolute 10 year probability of developing a fatal cardiovascular event[3]. Furthermore, treatment should be targeted to all medical and behavioural risk factors i.e. stimulating physical activity, aiding healthy food choices and encouraging non-smoking[3,10]. Online health advice instruments can be used as a tool to aid behavioural change[11]. The implementation of prevention programmes has to be cost effective. Reduction of cardiovascular risk factors seems to be cost effective [12-14]. To conclude, it is important to implement cost effective CVD prevention programmes to decrease CVD (risk factors) and associated costs[3]. The aim of the PreCardio-study is to determine the effects of a computer-tailored cardiovascular prevention programme with the general practitioner (GP) as a key figure. This prevention programme consists of interventions targeted at all risk factors with the use of information and communication technology (ICT) at different levels. At GP-level, a tool for computer-assisted cardiovascular risk screening and an informational website will be implemented. At patient-level, a personalised website with tailored advice will be foreseen.

## Methods/Design

### Study population

PreCardio is a randomized controlled trial. Approval was obtained from the ethics committee of the University Hasselt. A total of 350 subjects will be included in the study with a follow-up of 3 years. The inclusion criteria are age between 25–65 and insured by the Onderlinge Ziekenkas, insuring for guaranteed income in case of illness for self-employed. All study participants need to sign an informed consent and have access to the internet. Other eligitability criteria are not included.

### Recruitment

The **study participants **will be recruited through various channels. Firstly, all potential participants are insured by "De Onderlinge Ziekenkas". This membership gives the study secretariat access to necessary contact information. Secondly, all of the insured are self-employed, most of them lawyers. This provides the study office with two advantages: (1) communication is possible at the worksite and (2) the prevention programme is even more tailored to the uniform target group. The announcement of the study was already made through mass-media announcement (e.g. national newspapers, provincial journal and radio) but will be intensified at the worksites of the potential participants. Together with the involved Bars (of the province of Limburg in the East of Belgium) mass mailings and promotional activities at the worksite (e.g. information, fruit distribution,) will be organized. Thirdly, a large event will be organised for the participants of the intervention group and control group. Before joining these events a written informed consent, downloadable on a website with brief information, should be signed and mailed back to the study secretariat. At this large event general information about PreCardio will be given, a first screening and the ability to purchase health-related products.

The **general practitioners (GP) **that will participate in the study are GPs of the province of Limburg, situated in the East of Belgium. They will be recruited with involvement of their own provincial general GP platform "Limburgs Huisartsenplatform" and provincial institutions involved in health "LOGOs". Together with the GP platform and the LOGOs mass mailings to GPs of Limburg will be organised. Moreover, minimal two information sessions for GPs will be organised in the university building with information about the study and education about a new type of cardiovascular risk calculator will be offered. All participating GPs need to sign an informed consent and have access to the internet for downloading an electronic cardiovascular risk screenings programme based on the European Guidelines for CVprevention[3].

### Randomization

The study participants were randomized using a nonstratified randomization technique with a known probability. Each participant had a 67% chance to be allocated to the intervention group. The randomization was performed by an independent person. The names of the participants were written on papers that were put in sealed envelopes. Next, the envelopes were randomly assigned by hand to two baskets for the intervention and the control group, respectively, with a ratio of 2/1.

### Overall aim of the study

To examine the effects of medical and behavioural interventions on medical cardiovascular risk factors such as hypertension, hypercholesterolaemia and hyperglycaemia and behavioural risk factors such as a sedentary lifestyle, an unhealthy diet and smoking. Furthermore, the impact of changes in medical and behavioural risk factors on overall cardiovascular risk will be investigated.

#### Primary aims

1. To examine the effects of medical and behavioural interventions on medical parameters such as systolic blood pressure, diastolic blood pressure, total blood cholesterol level, blood glucose level (HbA1c), overweight and obesity, BMI.

2. To examine the effects of medical and behavioural interventions on health related behaviour such as physical activity; fat; fruit and vegetable intake; and smoking behaviour.

3. To examine the effects of medical and behavioural interventions on overall cardiovascular risk mediated by changes in medical and/or behavioural risk factors.

4. To determine the proportion study participants with a 10% reduction in systolic blood pressure with participants with hypertension

5. To determine the proportion study participants with a 10% reduction in diastolic blood pressure with participants with hypertension

6. To determine the proportion study participants with a 10% reduction in total cholesterol with patients with hypercholesterol

7. To determine the proportion study participants with a 10% reduction in HB1Ac with initial HB1Ac >7% (high risk)?

8. To determine the proportion study participants with a 10% reduction in weight with initial BMI >25 kg/m^2^?

#### Secondary aims

1. To determine the incidence of cardiovascular events

2. Number and total costs of cardiovascular events leading to loss of productivity

3. To examine the effects of medical and behavioural interventions on psychological constructs as stage of change, attitude towards behaviour, self-identity, perceived behavioural control, autonomous motivation, intention.

4. To examine the effects of medical and behavioural interventions on Health Related Quality of Life.

5. To examine the effects of social support on health related behaviour (physical activity, fat and fibre intake and smoking behaviour) and psychological constructs (stage of change, attitude towards behaviour, self-identity, perceived behavioural control, autonomous motivation, intention)

6. To perform a cost-effectiveness analysis of the medical and behavioural interventions using incremental cost-effectiveness ratios

### Design

**Figure 1:**
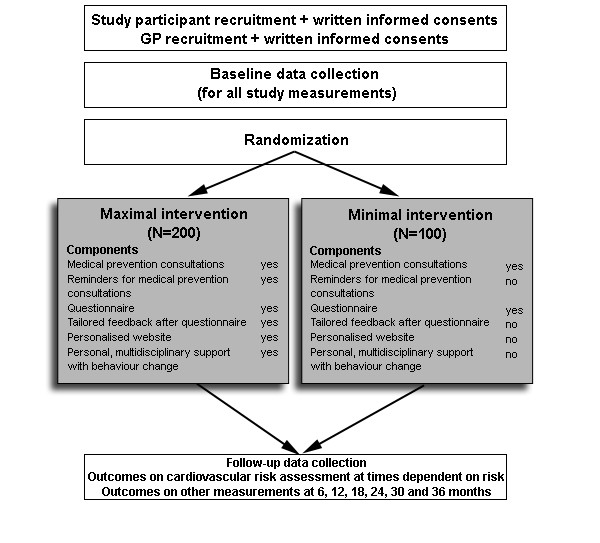
**PreCardio design**. GP: general practitioner.

**Figure 2:**
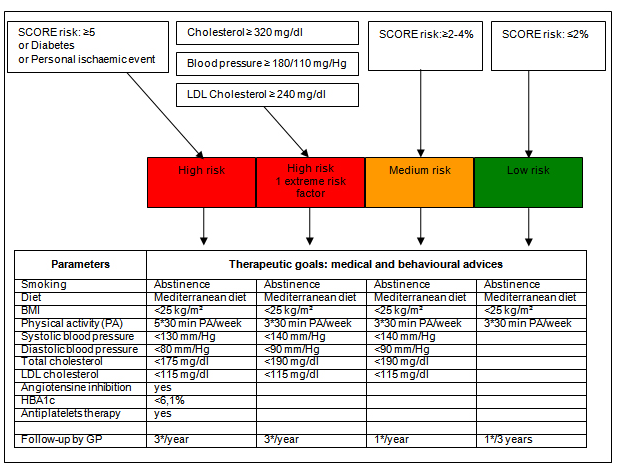
Adapted risk assessment flowchart [3, 16].

The intervention group as well as the control group receive a first medical prevention consultation with a GP of their choice. Fig 1 represents the study design. The GP performs a cardiovascular risk assessment using an adapted risk assessment flowchart (Fig 2). Medical data such as systolic and diastolic blood pressure, total blood cholesterol, LDL cholesterol (and HbA1c) are collected at this medical prevention consultation prior to risk determination. The intervention and the control group receive the therapeutic goals from the risk assessment flowchart. The intervention group receives supplementary tailored feedback regarding behaviour change based on the TransTheoretical Model or the Stages of Change Model[23]. Thus, the feedback is tailored to the stage of change the study participants are in for physical activity, diet and quit smoking. After the first medical prevention consultation all study participants get access to a website which includes a question list for baseline data collection. The intervention group receives tailored advice on this question list. The control group receives general advice to impede or avoid drop-out but with caution for causing bias. The question list includes items for physical activity assessment, fat intake assessment, fruit and vegetable intake assessment, smoking behaviour assessment and assessment of psychological variables. The physical fitness will be determined at the GP's office, at study participants home and at baseline data collection during the large event. For follow-up data collection these assessments will take place regularly. The intervention group receives the maximal intervention and the control group receives the minimal intervention.

### Study measurements

The study measurements in the PreCardio-study include cardiovascular risk assessment. Supplementary a questionnaire assessing physical activity; fat, fruit and vegetable intake; smoking behaviour and psychological variables will be filled out. Furthermore, physical fitness assessment will be performed. These assessments will take place at certain times during the 3 year follow up of the study (Table 1).

**Table 1:** Data collection

**Assessment of**	**Instrument**	**Means**	**0 m**	**4 m**	**6 m**	**8 m**	**12 m**	**18 m**	**24 m**	**30 m**	**36 m**
Cardiovascular risk (BMI, blood pressure, cholesterol, HBA1c)	Risk Calculator	GP	YES	~RISK	NO	~RISK	YES	NO	YES	NO	YES
Physical Activity (PA)	IPAQ	Website	YES	NO	YES	NO	YES	YES	YES	YES	YES
Fat intake	Questionnaire	Website	YES	NO	YES	NO	YES	YES	YES	YES	YES
Fruit & Vegetable intake	Food Frequency Questionnaire	Website	YES	NO	YES	NO	YES	YES	YES	YES	YES
Smoking behaviour	Questionnaire	Website	YES	NO	YES	NO	YES	YES	YES	YES	YES
HRQol	SF-36	Website	YES	NO	YES	NO	YES	YES	YES	YES	YES
Stages of change	Questionnaire	Website	YES	NO	YES	NO	YES	YES	YES	YES	YES
Attitudes	12 items	Website	YES	NO	YES	NO	YES	YES	YES	YES	YES
Self-identity	6 items	Website	YES	NO	YES	NO	YES	YES	YES	YES	YES
Perceived control over behaviour	3 items	Website	YES	NO	YES	NO	YES	YES	YES	YES	YES
Self-efficacy	6 items	Website	YES	NO	YES	NO	YES	YES	YES	YES	YES
Social Support	15 items	Website	YES	NO	YES	NO	YES	YES	YES	YES	YES
Physical fitness	Step test	Event/GP/home	YES	NO	NO	NO	YES	NO	YES	NO	YES

#### Cardiovascular risk assessment

**Figure 3:**
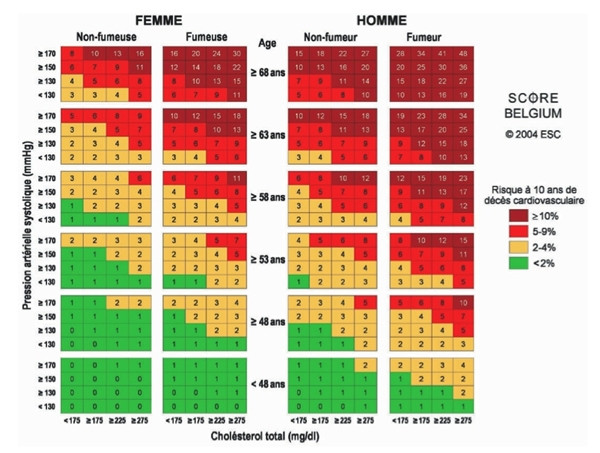
SCORE risk table [15].

Cardiovascular risk assessment will be based on the SCORE risk table (Fig 3) [15]. The overall cardiovascular risk score is the 10-year risk to die from CVD. According to SCORE a risk score of 4% or more is considered a high risk. A risk score in the range 2%–4% is considered a medium risk and a score lower than 2% is seen as a low risk[3]. The medical and behavioural advices following risk assessment are adapted from the Boland algorithm[16]. Cardiovascular risk assessment in the PreCardio-study is thus an adaptation of existing and validated risk assessment flowcharts in Belgium (Fig 2).

#### Physical activity assessment

For physical activity assessment it is important that each study participant gives an estimate of the intensity, duration and frequency of the physical activity he has in daily life. For this purpose a Dutch translation and computerized version of the International Physical Activity Questionnaire (IPAQ) was chosen[17]. This computerized version was found to be reliable and reasonably valid physical activity assessment tool for the general Belgian adult population[11].

#### Fat intake assessment and fruit and vegetable intake assessment

To stimulate rapid assessment only data collection will be limited to fat intake and fruit and vegetable intake. For fat intake assessment a computerized fat intake questionnaire with a good reliability and adequate validity was chosen[18]. For fruit and vegetable intake a short food-frequency questionnaire was used with a good test-retest reliability (Spearman *r *from 0.45 to 0.77), and adequate validity comparing the food-frequency questions with 7-day food records (Spearman *r *from 0.38 to 0.53)[19].

#### Smoking behaviour assessment

To obtain data about smoking behaviour, a short questionnaire regarding smoking from a national health questionnaire used by the federal government was selected[20]. This questionnaire includes questions for smokers as well as for former smokers.

#### Quality of life assessment

The Dutch translation of the Short Form 36 (SF-36) will be used to measure Health Related Quality of Life (HRQoL). This version was successfully tested in a Belgian and Dutch population with a Chronbach's alpha coefficient ranging from 0.81 to 0.91[21,22].

#### Assessment of psychological variables

##### Stages of change

Stages of change is a construct from the TransTheoretical Model[23]. The stages of change were assessed using a single table for assessing physical activity, diet and quitting smoking (Table 2). The formulation of the statements was based on the remarks about standardization in a recent meta-analysis[24] and questionnaires for measuring stages of change for physical activity[25] and fat intake[18].

**Table 2:** Assessment of stages of change

	30 minutes of moderate PA daily or 3*/week intensive PA	Low fat diet and 5 portions fruit and vegetables daily	Not smoking (if relevant)
I'm not performing the behaviour and I do not intend to in the next 6 months			
I'm not performing the behaviour but I plan to in the next 6 months			
I'm not performing the behaviour but I plan to in the next 30 days			
I perform the behaviour but no longer than 6 months			
I perform the behaviour but already longer than 6 months			

##### Attitudes towards behaviour change

Attitudes are an important element in the Theory of Planned Behaviour[26]. As recommended by Ajzen and Fishbein[27], a measure of attitude towards the target behaviour was obtained using 3 evaluative semantic differential scales. Study participants are asked to assign a value along a 7-point scale between opposing evaluative adjectives (e.g. pleasant-unpleasant, good-bad, relaxing-stressing)[28]. An example of an item requesting a value assignment for "To eat low fat every day for me is" along a 7-point scale between the opposing evaluative adjectives "pleasant" and "unpleasant".

##### Self-identity towards behaviour change

Self-identity is seen as the extent to which people see them fulfilling different societal roles[28]. In this study it is seen as the way people define themselves regarding a specific health behaviour (physical activity, a healthy diet and quitting to smoke). The question list includes 2 items for self-identity for each type of health behaviour. An item for physical activity is, for example, "I think of myself as someone who is physically active". An item for diet is, for example, "I am someone who often thinks about the importance of a healthy diet for my health". For smoking, an item for self-identity is, for example, "I am someone who thinks quitting to smoke is important". All were measured on 7-point Likert scales, anchored by strongly disagree-strongly agree.

##### Perceived control over behaviour

Perceived control over behaviour is related to self-efficacy, the two variables are significantly and highly correlated [29]. However, if the two variables are entered into a regression model for behavioural intention, they contribute independently to the model. Perceived behavioural control is usually measured in terms of difficulty or ease of performing the behaviour. This means that behaviours that are not under individuals' full volitional control are included [29]. Perceived control over behaviour is measured with 1 item. Participants indicate the extent to which they agreed with each item on 7-point Likert scales from strongly disagree-strongly agree. The items include "I have the feeling that daily 30 min moderate PA or 3 times sports a week is completely in my control the next month", and " I have the feeling that quitting to smoke is completely in my control the next month". The selection of these items was based on prior research with this concept regarding health-related food choice[28].

##### Self-efficacy

Self-efficacy is usually measured in terms of individuals' confidence in their ability to perform the behaviour [29]. Self-efficacy is measured with 2 items for each type of health behaviour. Participants rated these items using a 7-point Likert scale, anchored by strongly disagree-strongly agree. The items include "I am capable to adopt daily PA for 30 minutes or sport 3 times a week in the next month, also on days I feel bad, tired, stressed or depressed", "I think I am capable to adopt a low fat diet in the next month, also on days that I am very busy or family and friends request extra time from me" and "I am capable to quit smoking the next month, also on days I feel bad, tired, stressed or depressed". The items for self-efficacy were based on prior research with this concept regarding health-related food choice[28].

##### Autonomous motivation for behaviour change

People are autonomously motivated if they experience a true sense of volition and choice and act because of the personal importance of the behaviour [30,31]. If people are autonomously motivated the behaviour they perform is said to be autonomously regulated. Autonomous regulation is assessed with the Treatment Self-Regulation Questionnaire [31]. The Treatment Self-Regulation Questionnaire was used in former research on the effect of an intervention on diet and smoking cessation [31]. For assessing autonomous motivation for physical activity the Behavioural Regulation Exercise Questionnaire II (BREQ-II) was chosen for the measurement of autonomous motivation, with good psychometric properties[32,33].

##### Intention towards behaviour change

Intention to engage in a behaviour is a direct precursor to engaging in the behaviour itself [29]. Behavioural intention was assessed using 3 items: "I plan to adopt a healthy diet (low on fats and 5 portions of fruit or vegetables) in the next month", "I plan to adopt daily PA or 3 times sport a week in the next month" and "I plan to quit smoking in the next month". The 3 items could be answered using a 7-point Likert scale, with endpoints labelled strongly disagree-strongly agree. These items were based on previous research [30].

##### Social support

Social support is an important variable influencing behaviour change[34]. Consequently, several items were included in the question list to measure the amount and nature of experienced social support of the study participants. The answer possibility involved a 3-point Likert scale with following options: never/almost never, sometimes and often. The items included how often people feel encouraged by partner support, support of children older than 12, support of family or friends, support of colleagues, support by GP and support by the PreCardio-team for adopting a healthier diet, being more physically active and quitting smoking. Furthermore, study participants are asked if they have a fixed sports partner with answer possibilities: yes, sometimes and no.

#### Assessment of physical fitness

A step test will be used for assessment of physical fitness. The step test procedure used was modified from earlier procedures[35]. The step test consisted of stepping up and down a platform at a constant controlled by a metronome (90 beats per minute). Each beat initiates the movement of one leg up or down the platform. The stepping period lasted for 5 minutes. The height of the platform was determined according to patients' height and risk for complications (e.g. former personal ischemic event). For individuals smaller than 160 cm or with a former event a platform of 25 cm was used. For individuals with a height between 160 and 180 a platform of 33 cm was used. For people with a height above 180 cm a platform of 40 cm was used. The heart rate frequency was recorded between 1 minute and 1:30 minutes after completing the step test or stop because of exhaustion) in sitting position, between 2 minutes and 2:30 and between 3 minutes and 3:30. With a formula, the physical fitness level of the patient can be calculated. Few materials are needed in order to execute this test: a platform with a specific height, a stopwatch, and a metronome in order to determine the stepping speed.

### Interventions

#### Minimal intervention

##### Medical prevention consultation

The interventions targeted on medical parameters include a first medical prevention consultation with the GP. In this medical prevention consultation the cardiovascular risk (risk on having a heart attack in the next 10 years) will be calculated using a new computer programme. This computer programme is called "Electronic Prevention Record" (EPR). Innovative about the EPR is its connection to "Electronic Medical Record" (EMR). The EMR is also a computer programme, a record about the medical history and treatment provided to the patient. In the province of Limburg, the setting for the PreCardio-study, 75% of the GPs use an EMR[36]. In Belgium, 70% of the GPs use an EMR [37]. In Belgium there are 17 different providers of EMRs. The EPR generates, after a limited input (thanks to the connection to the EMR), the cardiovascular risk, the therapeutic goals (targeted on medical parameters and behaviour change) and a target date for the next follow-up consultation. This date is dependent on the determined cardiovascular risk. During these medical prevention consultations medical practice will be performed: measurement (e.g. blood pressure) and installation of treatment to influence medical parameters. Supplementary, the GP performs stage of change assessment (Table 2) using the EPR. The GP asks for PA and diet which statement fits the participant's situation best. If it is a smoker, the stage of change for quitting smoking is assessed as well. The stage of change assessment is for the control group not followed by tailored feedback for behaviour change.

**Figure 4:**
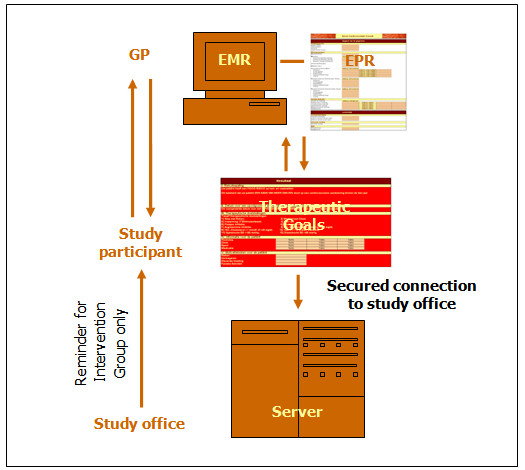
Data transfer.

All data collected at the medical prevention consultation are stored in the EPR that is connected to the EMR (Fig 4). Therapeutic goals in line with cardiovascular risk are determined. These goals, together with the data provided by the study participant are electronically sent to the study office by a secured connection. The information from the intervention group is closely examined and the participants of the intervention group will receive a reminder from the study office when it is time to plan their next medical prevention consultation.

##### Website with minimal information

The website available for the control group is limited and contains information about the minimal intervention (i.e. medical prevention consults *without *tailored feedback and *without *reminder from study office). It is also used as a means for study measurements at certain times during the 3-year follow up (Table 1). This limited website contains *general *feedback after the questionnaire with multiple assessments has been filled out by the study participants from the control group. This *general *feedback (= not tailored) is about physical activity, fat intake and smoking and is modified for different measurements in time to avoid drop-out. However, the advice will always be formulated very abstract to avoid 'contamination' of the control group as was the case in another multiple risk factor cardiovascular intervention study[38].

#### Maximal intervention

##### Medical prevention consultation + tailored feedback + reminder from study office

The maximal intervention includes the medical prevention consultation described under the minimal intervention. However, the maximal intervention includes two extra services in relation to the medical prevention consultation.

Firstly, supplementary to the therapeutic goals which are communicated to the patient, the intervention group receives tailored feedback according to the stage of change the study participant is in. For this purpose, the table to for stage of change assessment (Table 2) is included in the EPR. The GP asks for PA and diet which statement fits the participant's situation best. If it is a smoker, the stage of change for quitting smoking is assessed as well. Following this stage determination, the GP gets information about the stage of change the study participant is in for each behaviour, namely: precontemplation, contemplation, preparation, action or consolidation[23]. The tailored feedback that follows is also based on principles from Motivational Interviewing (MI) [39]. Tailored feedback following a classification in the contemplation stage for smoking gives the GP following information: "Accept and talk about the ambivalence of the patient regarding quitting to smoke"; "Talk with the patient about the advantages and disadvantages of smoking and quitting smoking. Don't give arguments to quit smoking yourself"; "Advise the patient to download the quitting to smoke leaflet on the personalised website"; "Advise the patient to look at step 1 and step 2 of the quitting to stop manual on the personalised website". Tailored feedback can be easily printed and given along with the participant by the GP. This printed tailored feedback for the participant following a classification in the contemplation stage for smoking contains following information: "You want to quit smoking and you know it could have advantages, but something keeps you from actually quitting"; "It can be a good idea to think about the advantages and disadvantages of smoking and quitting to smoke. The quitting to smoke manual (step 1 and step 2) on the website can be an aid in this process"; "You can also download a quitting to smoke leaflet". This kind of advice is given to every study participant in the intervention group for all relevant health behaviours.

Secondly, the intervention group receives the supplementary service of being reminded of their next follow-up prevention consult by the study office. As mentioned above, this target date is dependent on the cardiovascular risk of the participant. This reminder will be send by mail or the patient will be reminded by telephone.

##### Personalised Website with tailored feedback

**Figure 5:**
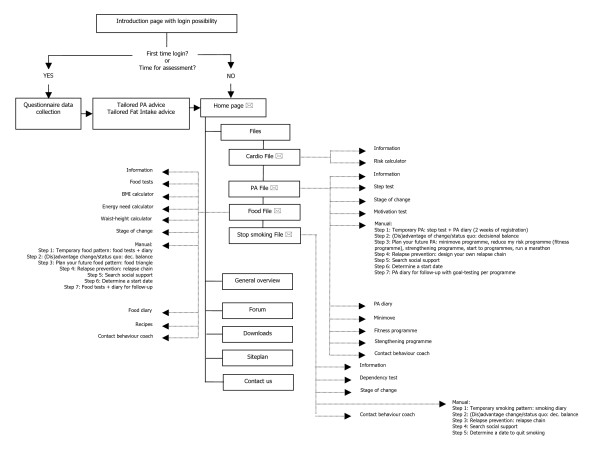
**Site plan of the personalised website (maximal intervention)**. PA: Physical Activity.

The intervention group gets access to a personalised website with tailored feedback based on questionnaires. Fig 5 shows the site plan of the personalised website. This website is supposed to aid people with behaviour change. The content is based on specific theories on behaviour change, such as the TTM[23,40], Self-Determination Theory (SDT) [30] and Theory of Planned Behaviour (TPB)[26]. Furthermore, operant learning principles (e.g. stimulus control, contingency management), principles from behavioural cognitive therapy (e.g. decisional balances, problem-solving techniques) and relapse prevention techniques are used as a basis for the content of the website, in particular for the manuals [40,41].

The personalised website contains tailored feedback after the questionnaire with multiple assessments has been filled out by the study participants. This tailored feedback regarding physical activity and fat intake is borrowed from previous research with proven efficacy and completely integrated in the website [11,18]. Moreover, more extensive tailored feedback on other assessment measures (e.g. stage of change, autonomous motivation for PA) is given in the different files on the website (PA file, Food File, Stop smoking File). In the General overview all activity from the patient and results are summarised. The General overview contains several extra's, namely: short links to the PA diary, Food diary and Stop smoking diary; results from medical prevention consultations and a personal calendar with important dates (target date for follow-up medical prevention consultation, start dates for behaviour change). On the homepage and in the Files (Cardio File, PA File, Food File and Stop smoking File) personal messages for each participant can be given. The message could be, for instance, an encouragement for behaviour change, an answer to a question of the participant or an announcement for an initiative of importance for the participant (e.g. group counselling for smoking-cessation). On the forum on the homepage participants can post messages, communicate with each other and ask questions to the PreCardio-team. The PreCardio-team guarantees answers concerning cardiovascular prevention and behaviour change. If the PreCardio-team can't provide an answer, other specialists will be consulted to provide an answer to the study participants. Of course, people can find all contact information of the PreCardio-team (study office) in the section "Contact us".

**Figure 6:**
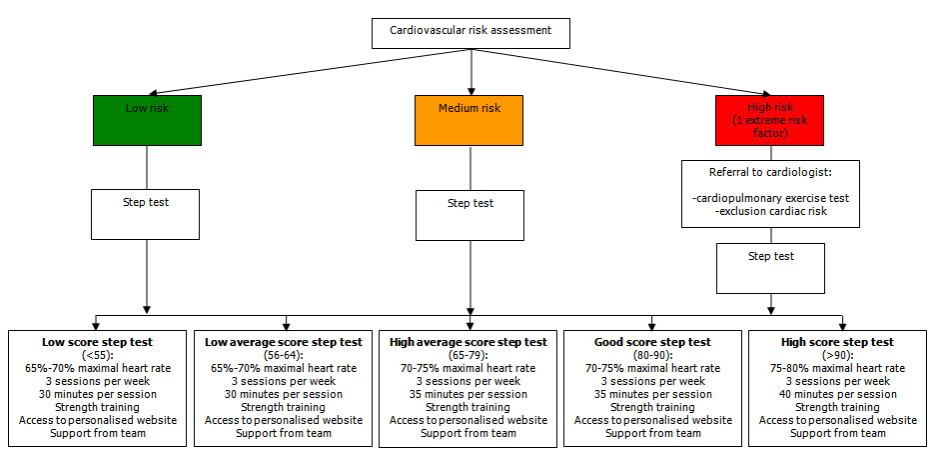
Algorithm for fitness programme "Reduce my risk" (maximal intervention).

The PA file contains several PA programmes with different difficulty levels. The easiest programme is "Minimove", this is a programme with mainly tips for improving PA through (Nordic) walking aiming at a health benefit. The programme most in line with the PreCardio mission, namely CVD prevention, is the "Reduce my risk" programme. This programme is a fitness programme aimed at improving physical fitness through more intensive PA. The basis for the fitness programme is a specially developed algorithm linked to the step test used in the study (Fig 6). Three different training programmes are advised, according to step test score determined by physical fitness assessment.

##### Support from multidisciplinary team

Next to the personalised website with tailored feedback, study participants of the intervention group are entitled to support from a multidisciplinary team. This multidisciplinary team includes: a cardiologist, a general practitioner, a psychologist, a physiotherapist and a dietician. Different media will be available for this personal contact: personal messages on the website, the forum on the website, e-mail, telephone and group sessions. The first types of media will be more frequently used than the latter, dependent on the stage of behaviour change patients are in. Moreover, the cost-effectiveness of the prevention programme must be guarded. The psychologist (= behaviour coach) can aid people more thoroughly with behaviour change, using the different manuals and behavioural (cognitive) therapy.

#### GP intervention

At GP-level a tool for computer-assisted cardiovascular risk screening (EPR) will be implemented. Minimal two information sessions for GPs will be organised in a university building with information about the study and education about the EPR. GPs will have access to an informational website. This informational website contains information about CVD prevention in general with a summary of the latest guidelines (also in a newsletter sent by mail). (Moreover, relevant articles will be provided to the GPs in PDF-format). Furthermore, it contains information about the PreCardio-study and the role of the GP in the study. On this website information about the EPR will be available (installation, how to use, background). GPs are provided with information on behaviour change, including video registrations of motivational interviewing adaptations for PreCardio. In the section on behaviour change more information will be given about the stages of change and type D-personality as a risk factor for CVD. The website for GPs also contains an overview of the personalised website for patients from the intervention group. GPs have access to a forum where they can post messages to open a debate with their participating colleagues about CVD prevention or the PreCardio-study or messages with a question for the PreCardio-team. In other sections contact information and links to relevant websites can be found.

### Analysis and power

A power calculation was performed to determine the number of study participants needed to detect a significant effect of the PreCardio prevention programme. A t-test will be used to compare the means of the outcome measures from the intervention and the control group. The sample size calculation is based on the population standard deviation of the primary outcome measure. For PreCardio, this is, for instance, systolic blood pressure. Kelley *et al *found a mean systolic blood pressure of 125 mm/Hg (SD 14 mm/Hg)[42]. The sample size calculation was performed with Nquery Advisor 4.0^®^. A two group t-test with a 0,05 two-sided significance level will have 82% power to detect the difference between a Group 1 mean of 120 mm/Hg and a Group 2 mean of 125 mm/Hg, a difference in means of -5 mm/Hg, assuming that the common standard deviation is 14 mm/Hg, when the sample sizes in the two groups are 200 and 100, respectively (a total sample size of 300). Other outcome measures were also used to determine the necessary sample size. A recent study about the effect of a computer-tailored intervention to reduce fat intake included at post-test a mean fat intake of 85 grams/day (SD 34.5 grams/day) [43]. A two group t-test with a 0,050 two-sided significance level will have 80% power to detect the difference between a Group 1 mean of 85 grams/day and a Group 2 mean of 97 grams/day, a difference in means of -12 grams/day assuming that the common standard deviation is 34.5 grams/day, when the sample sizes in the two groups are 200 and 100, respectively (a total sample size of 300). Another study evaluating a website-delivered computer-tailored intervention for increasing physical activity increased physical activity with a mean of 77 minutes in the intervention group [44]. In this study the total moderate- and vigorous-intensity physical activity was chosen as an outcome measure. This measure can be used to determine the necessary sample size to detect a difference in means in the present study. At follow up this measure had a mean of 363 minutes (SD 323) for the intervention group and 241 minutes (SD 269) for the control group [43]. A two group t-test with a 0,05 two-sided significance level will have 86% power to detect the difference between a Group 1 mean of 363 minutes and a Group 2 mean of 241 minutes, a difference in means of 122 minutes, assuming that the common standard deviation is 323 minutes, when the sample sizes in the two groups are 200 and 100, respectively (a total sample size of 300).

A linear regression model will be employed to assess the effects of the prevention programme on change in overall cardiovascular risk from baseline to 3 year follow-up.

### Cost and cost effectiveness analysis

#### Cost analysis

Next to an effect evaluation a cost analysis will be performed. There are different costs that will be measured in the PreCardio-study: (1) One-time costs, this is the total cost for the implementation of the prevention programme (e.g. construction costs of personalised website); (2) Costs for medical resource use (e.g. drug costs, laboratory costs, primary prevention consults); (3) Continuous costs: (acute care) costs for CVD, costs for CVD morbidity and mortality, hospitalization costs (4) Administration costs; (5) Personnel costs (PreCardio-team, other professionals); (6) Travelling and time costs (e.g. study participant's time and GP time); (7) Indirect costs due to productivity loss; (8) Material costs and overhead. This study will be performed from a health service perspective.

#### Cost effectiveness analysis

In cost effectiveness analysis incremental cost-effectiveness ratios (ICER) are used as an economic outcome measure. An ICER can be calculated as incremental cost divided by incremental effectiveness [45]. Incremental cost is calculated as total cost for the treatment programme minus total cost for the comparison programme. Incremental effectiveness can be calculated as Quality Adjusted Life Years (QALYs) gained by the treatment programme minus QALYs gained by the comparison programme. This is life expectancy adjusted by utility, a measure of quality of life[46]. In the PreCardio-study ICERs will be determined. A specific type of cost-effectiveness analysis, a cost-utility analysis, will be performed, using QALYs as a measure of effects. Inspired by a comparable study of Munro *et al*., SF-36 data were converted using a specific algorithm into health state utility values for the determination of incremental QALYs[47,48].

## Discussion

The PreCardio-study is a randomized controlled trial. However, the shared worksite setting could lead to an intervention contamination. In this regard, the failure to find significant effects from the Heartbeat Wales programme due to contamination of the reference area is relevant[38]. Partially, this study is similar to the PreCardio-study since it compares the effects of a multiple risk factor intervention. However, the multiple risk factor intervention from the "Heartbeat Wales" programme was less individually tailored, didn't include GPs as key figures, offered no multidisciplinary support and made no use of modern information technology like a personalised website for participants of the study. The benefits of these interventions targeted at behavioural risk factors could be substantial. After all, the PREMIER study, a clinical trial of comprehensive lifestyle modification for blood pressure control, was already effective in reducing cardiovascular disease risk [49]. This study is similar to the PreCardio-study because it targets one of the medical risk factors for cardiovascular disease. However, in the present study other risk factors will be targeted as well leading to a reduction in the overall cardiovascular risk.

Several aspects of the PreCardio-study are noteworthy. Firstly, all interventions are based on recent guidelines and knowledge on CVD prevention. Moreover, this knowledge was taken as a basis to develop a new computer programme for GPs. After limited input, this programme automatically determines CVD risk and generates therapeutic goals according to this risk. Furthermore, tailored advice is provided to the study participant. The involvement of GPs in the PreCardio-study is paramount. Secondly, study participants are provided with a number of aids to support them with behaviour change. These include a personalised website with tailored feedback and personal support from a multidisciplinary team. Consequently, a reduction in medical and behavioural risk factors resulting in a reduction in overall CVD risk is expected.

## Abbreviations

BMI, body mass index; BREQ-II, Behavioural Regulation Exercise Questionnaire II; CVD, cardiovascular diseases; EMR, electronic medical record; EPR, electronic prevention record; GP(s), general practitioner(s); HRQoL, Health Related Quality of Life; ICER, cost-effectiveness ratio; ICT, information and communication technology; IPAQ, International Physical Activity Questionnaire; MI, motivational interviewing; PA, physical activity; QALY, Quality Adjusted Life Year; SDT, Self-Determination Theory; SF-36, Short Form 36 TBP, Theory of Planned Behaviour.

## Competing interests

The author(s) declare that they have no competing interests.

## Authors' contributions

The PreCardio-study Protocol was written by prof dr Neree Claes (NC) and psychologist Nele Jacobs (NJ). Both authors have made substantial contributions to this protocol and performed a feasibility study prior to the development of this study protocol. NC, project manager, has elaborated the study design and co-authorised the manuscript. NJ drafted the manuscript and participated in the study design. Both authors read and approved the final manuscript.

## Pre-publication history

The pre-publication history for this paper can be accessed here:


